# Effects of different mulching practices on soil properties and soil microbial communities in tomato production

**DOI:** 10.3389/fmicb.2025.1734062

**Published:** 2026-02-03

**Authors:** Xiaoxia Li, Jinjun Cao, Dan Li, Kunpeng Jin, Yongzhong Liu, Wanxing Li

**Affiliations:** 1 Millet Research Institute, Shanxi Agricultural University, Changzhi, China; 2 Key Laboratory of Sustainable Dryland Agriculture of Shanxi Province, Taiyuan, China

**Keywords:** mulching, soil bacteria, soil fungi, soil properties, tomato yield

## Abstract

**Introduction:**

Agricultural mulches are commonly used for their benefits, however, the mechanisms by which they affect microbial communities to mediate soil properties that influence tomato growth remain unclear.

**Methods:**

A three-year experiment was conducted comparing four treatments: plastic film mulching alone (SBF), straw mulching alone (SM), film-straw dual mulching (SBFSF), and no mulching (CK). Their effects on soil properties, microbial communities, and tomato growth were systematically evaluated.

**Results:**

All mulching treatments significantly increased tomato yield, with SBF, SM, and SBFSF demonstrating improvement of 32.87%, 22.17%, and 50.17%, respectively. SBFSF exhibited the greatest dry matter weight (shoot plus root), root length, and root surface area 40 days post-transplanting, and it showed the strongest effect on soil moisture and thermoregulation. SM and SBFSF significantly increased soil organic carbon (SOC: +5.92%, +4.04%), total nitrogen (TN: +6.34%, +4.46%), available potassium (AK: +18.15%, +10.91%), and available phosphorus (AP: +2.60%, +2.15%). SBFSF significantly reduced the diversity of soil bacteria and fungi, howerer, it selectively increased the relative abundances of plant growth-promoting rhizobacteria and functional microorganisms involved in carbon-nitrogen cycling, such as the bacterial phylum Firmicutes(+125.18% to +193.55%), genera *Lysobacter* (+49.19% to +186.62%) and *Bacillus* (+168.56% to +273.00%), and fungal phyla *Ascomycota* (+7.99% to +8.19%) and *Mortierellomycota* (+11.05% to +98.71%), including the genus *Trichocladium* (+30.43% to +269.95%). In contrast, SM and SBF led to an increase in the abundance of pathogenic fungi (*Fusarium*, *Cladosporium*, *Alternaria*, and *Cephaliophora*), elucidating their inferior yield performance compared to SBFSF.

**Discussion:**

Partial least squares path modeling (PLS-PM) analysis indicated that mulching practices directly and positively influenced the soil bacterial and fungal community composition and negatively affected soil fungal community diversity, which indirectly effecting tomato growth by modulating soil properties. These results provide a scientific foundation for improving mulching, and sustainable agricultural practices.

## 1 Introduction

Mulching practices play a crucial role in tomato cultivation for regulating soil hydrothermal conditions and the soil micro-environment (Chakraborty et al., 2008; Wróblewska et al., 2019). The predominant mulching methods include plastic film mulching, straw mulching, net mulching with varying aeration, and biodegradable mulching films made from different materials. Plastic film mulching, net mulching, and biodegradable mulching films modify the surface energy balance and hydrothermal exchange, thereby promoting crop growth (Zhao S. Q. et al., 2023). Conversely, Straw mulching (SM) reduces the need for chemical fertilizers, increases soil organic matter, improves soil structure, and facilitates nutrient cycling (Wang et al., 2023; Hu et al., 2024; Du, 2024). Although mulching benefits are well documented and several studies have examined links among mulching, soil properties, microbial communities, and crop growth (Luo et al., 2025; Han et al., 2024), most work has evaluated a single mulching type. Few studies have systematically assessed the synergistic effects of a double mulching system (plastic film combined with straw) on soil microorganisms, soil physical and chemical properties, and crop performance. In particular, under specific regional soil and climatic conditions, the mechanisms by which combined plastic-film and straw mulching alters the soil environment and crop growth through regulation of key microbial groups remain unclear.

Soil microorganisms play an important role in soil (Philippot et al., 2023), and their community composition, diversity and function directly affect soil fertility and plant health. Previous studies have shown that various mulching techniques can affect soil microbial communities by regulating the soil microenvironment, such as soil temperature, moisture, aeration, and carbon and nitrogen cycling, thereby potentially change the structure, diversity and function of soil microbial communities (Dang et al., 2025; Dai et al., 2024). Studies have demonstrated that straw mulching can provide abundant carbon sources that promote the growth of bacterial and fungal biomass and the activity of beneficial functional bacteria involved in carbon-nitrogen cycling (Wang et al., 2024). However, the application of plastic mulches may lead to niche differentiation within microbial communities by creating distinct microenvironments, potentially favoring the growth of certain pathogenic bacteria (Qi et al., 2022). Moreover, film-straw dual mulching has been shown to increase the abundance of straw-degrading bacteria, elevate soil organic content, enhance nutrients availability, and consequently improve crop yields (Yang et al., 2024). However, the impacts of different mulching practices on the soil microenvironment vary significantly. The extent to which this difference, in particular selectively impacts the abundance and activity of beneficial (for instance plant rhizosphere growth promotors) or pathogenic bacteria, and how rhizospheric microorganisms react on this additional disturbance as well interact in one coherent system with soil nutrient dynamics in order to ultimately control growth and yield of crops, is an intriguing scientific question that should be resolved by present studies. In particular, the integrated film-straw dual mulching (SBFSF) method, which theoretically combines the advantages of both individual practices, lacks systematic empirical research regarding its comprehensive effects on soil microbiota and properties, as well as the underlying mechanisms.

Tomato, a crucial vegetable crop globally, is commonly cultivated under greenhouses using mulching technology to enhance growth conditions (Gu et al., 2020). This study, based on a three-year experiment, investigates the impact of different mulching practices on soil properties and soil microbiology in tomato production. The specific objectives were: (1) to compare the impacts of film-straw dual mulching (SBFSF), plastic film mulch alone (SBF), straw mulch alone (SM), and no mulch (CK) on tomato plant growth and yield; (2) to examine changes in soil physical and chemical properties, and microbial communities under different mulching practices; and (3) to elucidate the mechanisms through which soil bacteria and fungi drive soil factors to promote tomato growth. The findings of this study are expected to provide a solid scientific basis and practical guidance for optimizing mulching techniques in tomato cultivation, steering the soil micro-environment, and harnessing the functional potential of soil microbes, thereby contributing to green and sustainable agricultural development.

## 2 Materials and methods

### 2.1 Site description and experimental design

The experiment was carried out at the Jindong Organic Dryland Farming Experimental Demonstration Base of Shanxi Agricultural University in Dianshang Town, Huguan County, Changzhi City, Shanxi Province (E113°27′, N36°03′), within a steel-framed arched plastic greenhouse (90 m long, 8 m wide, 3.5 m high at the ridge, and 1.8 m high at both sides, with natural ventilation). This site has a warm temperate semi-humid continental monsoon climate, with an average annual temperature of 10 °C and rainfall of 574.5 mm. The experiment was conducted in a completely randomized design with four treatments: no mulching (CK), straw mulching (SM, whole straw was covered on the ridges, 650 kg·ha^−1^), plastic film mulching (SBF), and combined film-straw mulching (SBFSF, plastic film on ridges with straw in furrows, 650 kg·ha^−1^). Each treatment had three replications, and every plot with five beds and five furrows. Planting specifications included two rows per bed, 35 cm inter-plant spacing, 40 cm inter-row spacing, 60 cm ridge width, 80 cm furrow width, and 22 tomato plants per row. Drip irrigation lines were placed along the planting rows. Tomato seedlings were transplanted on May 22, 2024, and the vines were removed on September 15, 2024. Standard agronomic practices, such as irrigation, pruning, and pesticide application (Supplementary Table S1), were uniformly applied to all treatments. The tomato variety tested was “*Rirun No. 8*,” utilizing 800 mm wide, 0.01 mm thick silver-black plastic film and corn straw from the previous year’s harvest at the site for mulching. The initial soil physicochemical indicators at 0–20 cm soil layer are provided in Table 1.

**Table 1:** Key properties of the experimental soil at 0–20 cm.

Soil layer	SOC	Total nitrogen	Available phosphorus	Available potassium	Bulk density	pH
(cm)	(g·kg^−1^)	(g·kg^−1^)	(mg·kg^−1^)	(mg·kg^−1^)	(g·cm^−3^)	
0–20	33.2	1.4	13.42	133.42	1.25	8.20

### 2.2 Sampling and measurement

#### 2.2.1 Soil sample collection

**Figure 1:**
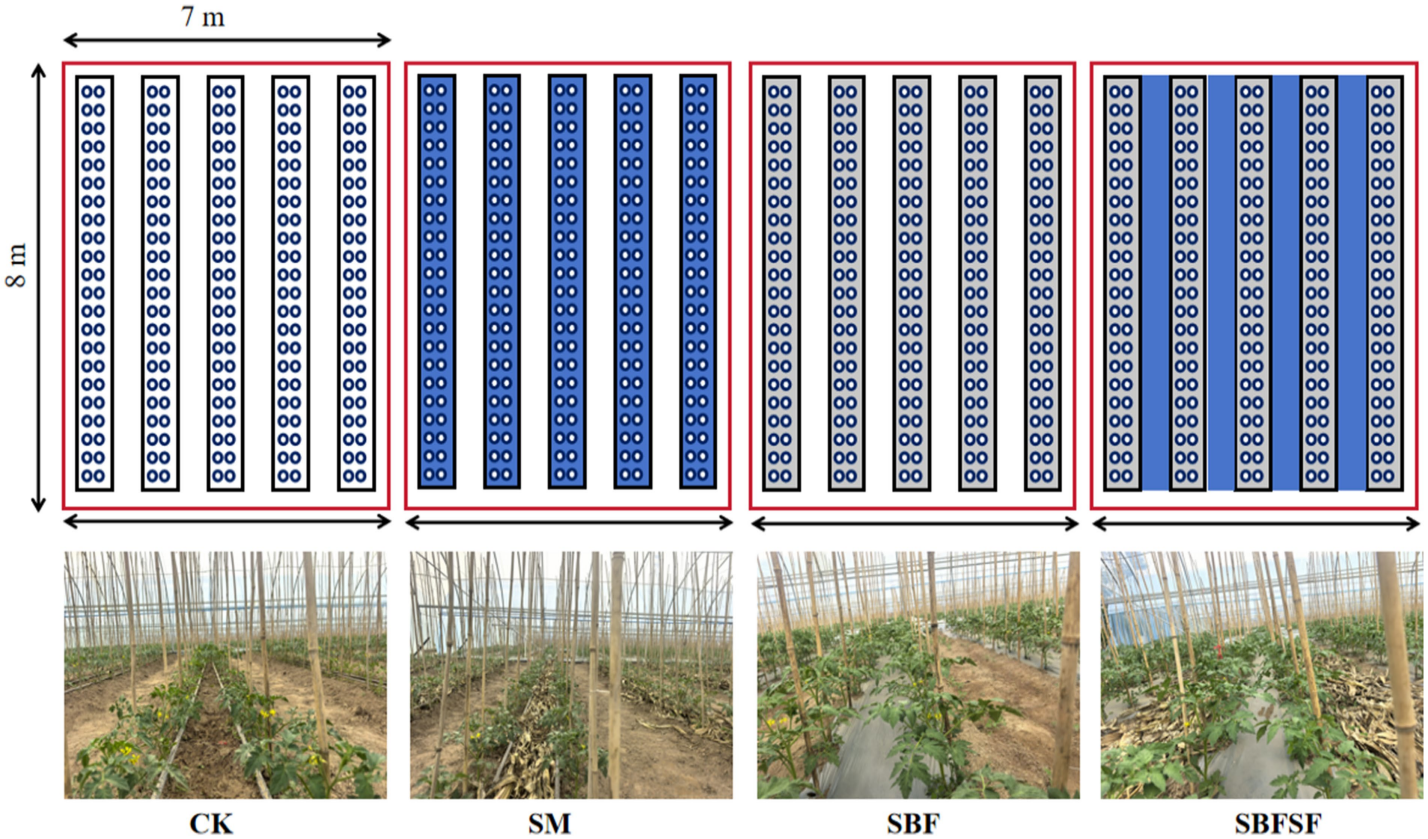
Schematic diagram showing the fields conducted with four treatments.

To investigate the underlying mechanism by which mulching practices influences soil fertility and soil microbial community structure to improve tomato plant growth over a 3-year period, soil samples were collected in early September 2024 before tomato uprooting at the end of the experiments. The rhizosphere soil from five tomato plants was shaken off and mixed to form composite soil samples for each treatment, then passed through a 2 mm sieve to remove gravel and residual roots. The soil samples were divided into two portions: one for analyzing soil chemical characteristics and the other stored at −80 °C for microbial DNA extraction (see Figure 1).

#### 2.2.2 Soil physical and chemical properties measurement

Soil moisture content was assessed every 20 days (avoiding periods of drip irrigation) by sampling using a 1 m soil drill along the tomato rows, extracting soil samples at 20 cm depth intervals from the surface to 1 m. The samples were then oven-dried at 105 °C until a constant weight and finally calculated the average gravimetric soil moisture content (g water/g dry soil) for the composite 1 m depth sample. Soil bulk density was determined at harvest using the ring knife method, and he cutting ring had an inner diameter of 70 mm and a height of 52 mm. Soil temperature was recorded every 2 h with a USB temperature logger (Jingchuang RC-5, Jiangsu Jingchuang Electric Co., LTD.), buried at depths of 10 cm and 20 cm within the tomato planting rows.

Soil organic carbon (SOC) was determined by digestion with 0.8 M K_2_Cr_2_O_7_ and concentrated H_2_SO_4_, followed by titration of residual dichromate (titration was performed using o-phenanthroline as the indicator). Total nitrogen (TN) was measured using an elemental analyzer (Vario EL III, Elementar, Germany). Available potassium (AK) was extracted with 1 M NH₄OAc (pH 7.0) and measured by flame photometry. Available phosphorus (AK) was extracted with 0.5 M NaHCO₃ (pH 8.5) and determined by the molybdenum blue method, then quantified via molybdenum-antimony anti-UV spectrophotometric colorimetry (Bao, 2000). The soil pH value was measured by a calibrated pH meter (FE28-Standard, Mettler-Toledo, Switzerland, soil-to-water ratio was 1:2.5).

#### 2.2.3 Soil DNA extraction and high-throughput sequencing

In this study, total DNA of soil bacteria and fungi was isolated using the OMEGA Soil DNA Kit (D5635-02) (Omega Bio-Tek, Norcross, GA, USA). Subsequently, the V3-V4 hypervariable regions of the bacterial 16S rRNA gene and the fungal ITS1 region were PCR amplified. The amplification products were electrophoresed on a 2% agarose gel, and target DNA fragments were recovered using the Axygen DNA Gel Extraction Kit. The PCR products were quantified with the Quant-iT PicoGreen dsDNA Assay Kit on a Microplate Reader (BioTek, FLx800). Library preparation and high-throughput sequencing were conducted by Personal Biotechnology Co., Ltd. The raw reads were deposited in the National Genomics Data Center, China National Center for Bioinformation (CRA037497).

### 2.3 Determination of dry matter weight of tomato plants, root morphology, and yields

The dry weight and root morphology in the 0–20 cm soil layer were monitored every 20 days post transplantation of the tomato plants. Following this, the tomato plants were brought back to laboratory, where their dry weights were quantified after 105 °C of exposure for 30 min, drying them in an oven at 80 °C until the constant weight and weighing the samples (Wang F. et al., 2021). The root system determination method according to the following procedure: a custom-designed root extractor (in a cube shape, 20 cm × 20 cm × 20 cm, 3 mm thick, with one side shaped like a knife to facilitate soil penetration) was inserted into the soil at the tomato root location and driven in using a hammer after removing the aboveground portion of the plant. Subsequently, the root-soil block was then excavated with a spade and trimmed neatly using a professional cutting tool to obtain a standardized sample. The excavated root system along with the soil was transported to the laboratory, then carefully washed off the soil adhering to the roots. The root system was then submerged in a glass tank filled with water, where the roots were carefully separated and scanned using an LA2400 root scanner (Canadian Reagent Instruments Company) to collect data on total root length and root surface area. Finally, the scanned roots were then oven-dried at 80 °C to a constant weight for calculating the dry matter weight of the root biomass.

During the harvest seasons of 2022–2024, tomatoes were harvested in batches from the two middle rows of each plot, and the yield was calculated on a per-hectare basis.

### 2.4 Data processing and analysis methods

The data were organized using Microsoft Excel 2007 (Microsoft Corp., USA), and variance analysis was performed with SPSS 27.0 (SPSS Inc., Chicago, USA), employing Duncan’s method for significance testing, including tomato yield, dry matter weight, root morphology, soil physical and chemical properties as well as soil bacterial and fungal diversity index (all data were subjected to tests for normality and homogeneity of variances, and for datasets that did not meet the assumptions of normal distribution or homoscedasticity, appropriate data transformations were applied). Principal coordinates analysis (PCoA) was conducted using the Bray-Curtis distance matrix algorithm to compare the taxonomic composition among treatments. Functional prediction of microbial communities was conducted using PICRUSt2 software. Redundancy analysis (RDA) based on a linear model was performed to assess the effects of soil physical and chemical properties on the structure of soil bacterial and fungal communities. Partial least squares path model (PLS-PM) was used to explore relationships among soil physical properties, soil chemical properties, soil bacteria, soil fungi and tomato plant. Both RDA and PLS -PM analyses were implemented using the vegan and plspm packages in R language, respectively. Figures were generated using Origin 2022.

## 3 Results

### 3.1 Tomato yields, dry matter weight and root morphology

#### 3.1.1 Tomato yields

**Figure 2:**
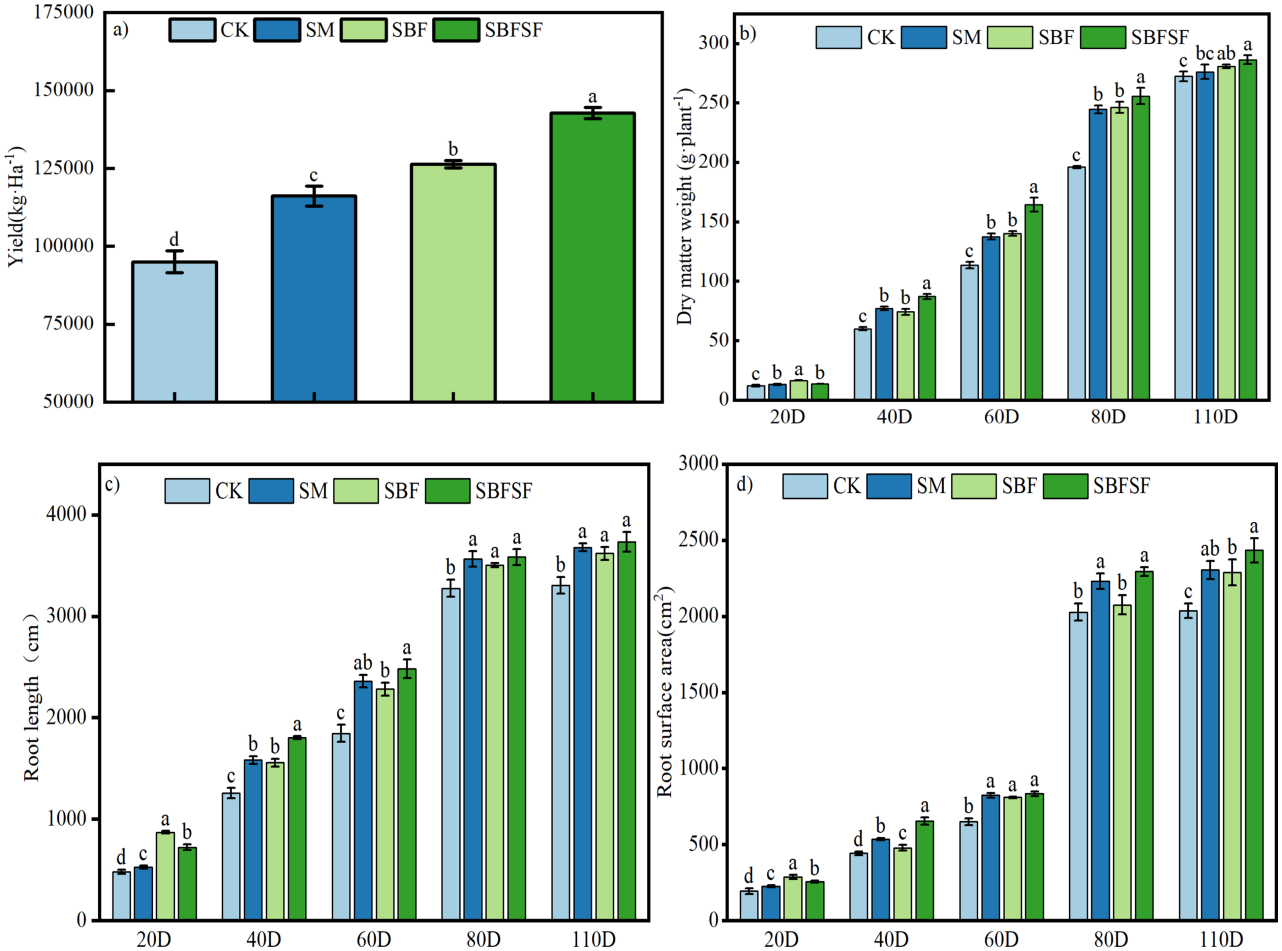
Tomato yield **(a)**, dry matter weight **(b)**, and root morphology **(c,d)** under different mulching practices.

The results showed significant yield increases in all mulching treatments (SM, SBF, SBFSF) over the control during 2022–2024 (SM vs. CK: *p* = 0.010; SBF vs. CK: *p* = 0.029; SBFSF vs. CK: *p* < 0.005), with three-year average gains of 17.11% ± 3.12%, 24.14% ± 7.53%, and 35.36% ± 7.82%, respectively. The annual increases were 11.42% (*p* < 0.001), 9.15% (*p* < 0.01), and 23.62% (*p* < 0.001) in 2022 (Supplementary Figure S1) 17.74% (*p* < 0.001), 30.40% (*p* < 0.001), and 32.30% (*p* < 0.001) in 2023 (Supplementary Figure S1); and 22.17% (*p* < 0.001), 32.87% (*p* < 0.001), and 50.17% (*p* < 0.001) in 2024 (Figure 2a).

#### 3.1.2 Dry matter weight of tomato plants

In all stages of growth, the dry matter weight of plants under each mulching treatment were significantly higher than that of the control. Specifically, SM, SBF, and SBFSF showed an average increase of 14.54% (SM vs. CK: *p* > 0.05), 19.19% (SBF vs. CK: *p* < 0.001), and 23.44% (SBFSF vs. CK: *p* < 0.001), respectively, compared to CK. At 20 days post-transplanting, the dry matter weight under SBF was significantly greater than that under SM and SBFSF, with no significant difference between SM and SBFSF. By 40 days post-transplanting, SBFSF exhibited the highest dry matter weight, with no significant difference between SM and SBF (Figure 2b).

#### 3.1.3 Root morphology

Throughout the growth period, all mulching treatments demonstrated superior root length and surface area compared to CK. On average, SM, SBF, and SBFSF showed increases in root length of 13.85% (SM vs. CK: *p* < 0.001), 24.18% (SBF vs. CK: *p* < 0.001), and 25.09% (SBFSF vs. CK: *p* < 0.001), respectively, and in root surface area of 14.53% (SM vs. CK: *p* < 0.001), 15.76% (SBF vs. CK: *p* < 0.001), and 23.51% (SBFSF vs. CK: *p* < 0.001), respectively, relative to the control. Specifically, at 20 days post-transplanting, SBF exhibited significantly greater root length and surface area compared to SM and SBFSF. By 40 days post-transplanting, SBFSF displayed the highest values for both root length and surface area, with no significant difference observed compared to SM by 60 days (Figures 2c,d).

### 3.2 Soil physical properties

#### 3.2.1 Soil water content

**Figure 3:**
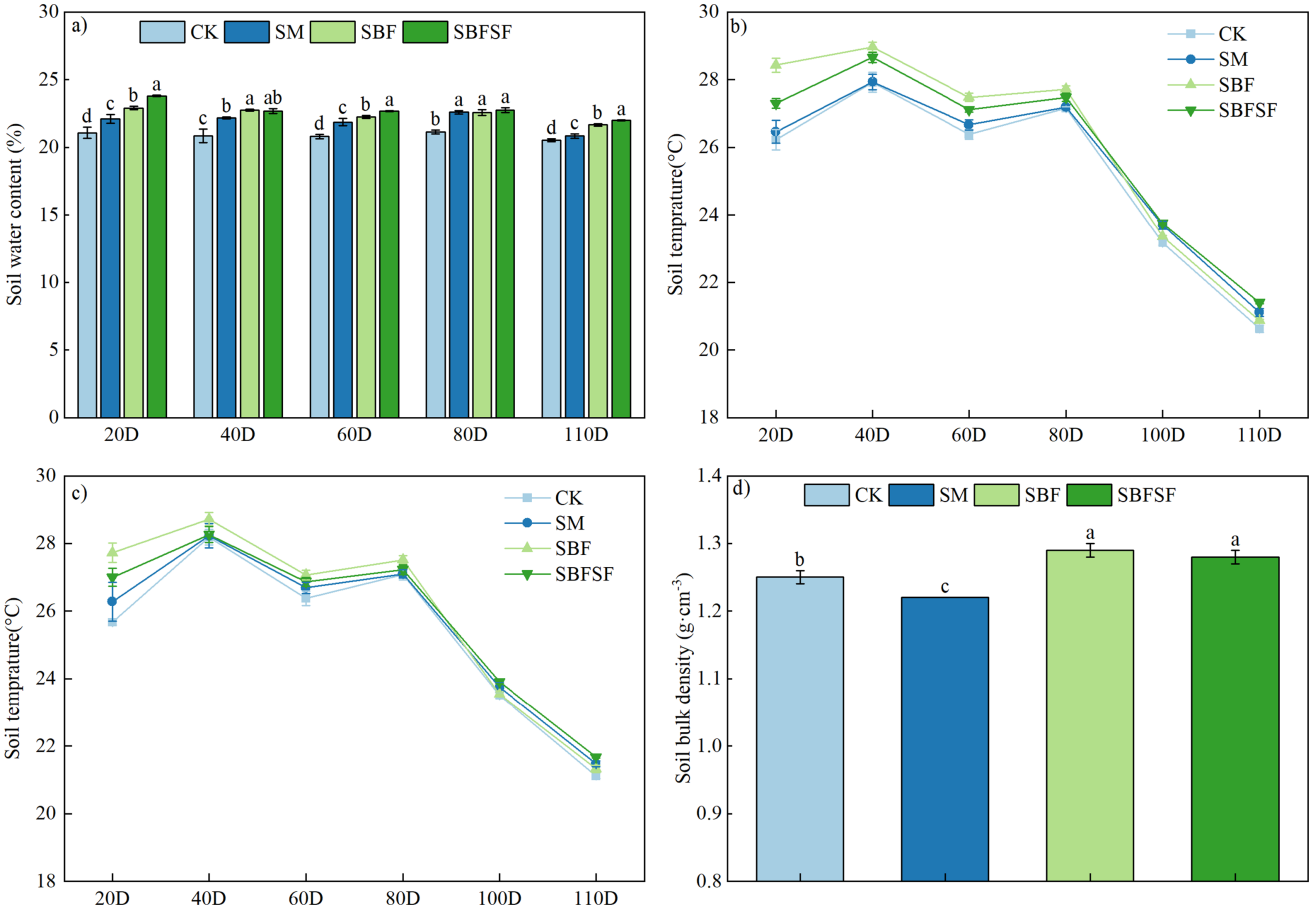
Soil water content, soil temperature, and soil bulk density under different mulching practices: **(a)** Soil water content; **(b)** soil temperature at 10 cm; **(c)** soil temperature at 20 cm; **(d)** soil bulk density.

At 20, 60, and 110 days post-transplanting, soil moisture content under the mulching treatments consistently exhibited the following order: SBFSF > SBF > SM > CK. The three mulching treatments increased soil moisture by 4.89–12.9%, 5.14–9.04%, and 1.51–7.16%, respectively, compared to CK. At 40 days after transplanting, SBF showed the highest moisture content, though no significant difference was observed compared to SBFSF. At 80 days after transplanting, there were no significant differences among the three mulching treatments, yet all maintained higher moisture levels than CK (Figure 3a).

#### 3.2.2 Soil temperature

Soil temperature rose at the beginning of tomato growth period, and then declined with the slower fluctuations under deeper soil layer (Figures 3b,c). All mulching treatments maintained higher soil temperatures compared to the control (CK), with temperature increases ranging from 0.01–2.21 °C at 10 cm depth and 0.02–2.05 °C at 20 cm depth. The thermal dynamics displayed distinct temporal patterns: during the first 80 days after transplantation, soil temperatures followed the order: SBF > SBFSF > SM > CK. From 80 to 110 days, the temperature pattern shifted to: SBFSF > SM > SBF > CK. In contrast to SBF, both SBFSF and SM treatments demonstrated thermoregulatory effects, providing cooling during high-temperature periods while maintaining warmth during cooler periods. SBFSF had the least soil temperature fluctuation over the full growth period of tomato. Correlation analysis showed no relationship between soil moisture and temperature at 20, 40, 60, and 80 days after tomato transplanting, but a strong relationship emerged at 110 days after transplanting, with a correlation coefficient of 0.966 (*p* < 0.001).

#### 3.2.3 Soil bulk density

The soil bulk density decreased by 2.40% (*p* < 0.01) in the SM treatment compared to the CK treatment, whereas the SBF and SBFSF treatments demonstrated notable increases of 3.20% (*p* < 0.01) and 2.40% (*p* < 0.01), respectively (Figure 3d).

### 3.3 Soil chemical properties

**Figure 4:**
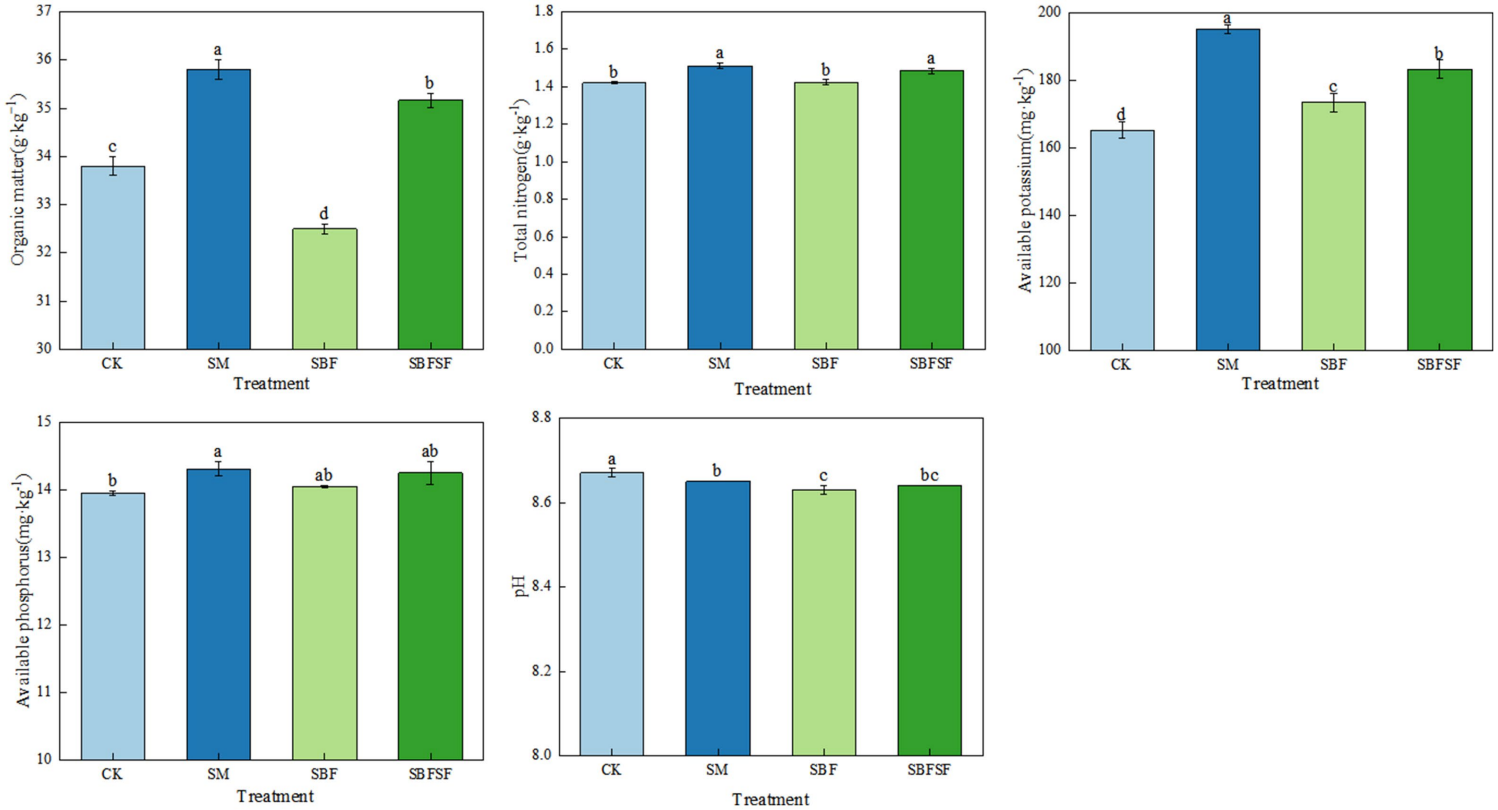
Characteristics of soil chemical properties under different mulching practices.

Soil chemical properties exhibited significant change under different mulching treatments after 3 years of tomato cultivation (Figure 4). Compared to the control (CK), SM and SBFSF significantly increased the contents of soil organic matter, total nitrogen, available potassium, and available phosphorus, with corresponding increases of 5.92%, 6.34%, 18.15%, and 2.60% (*p* < 0.01) for SM, and 4.04%, 4.46%, 10.91%, and 2.15% (*p* < 0.01) for SBFSF. In contrast, SBF resulted in a significant reduction in soil organic matter content by 3.85% (*p* < 0.01), though it increased available potassium content by 4.98% (*p* < 0.01). All mulching treatments significantly reduced soil pH.

### 3.4 Microbial communities and potential metabolism functions

**Figure 5:**
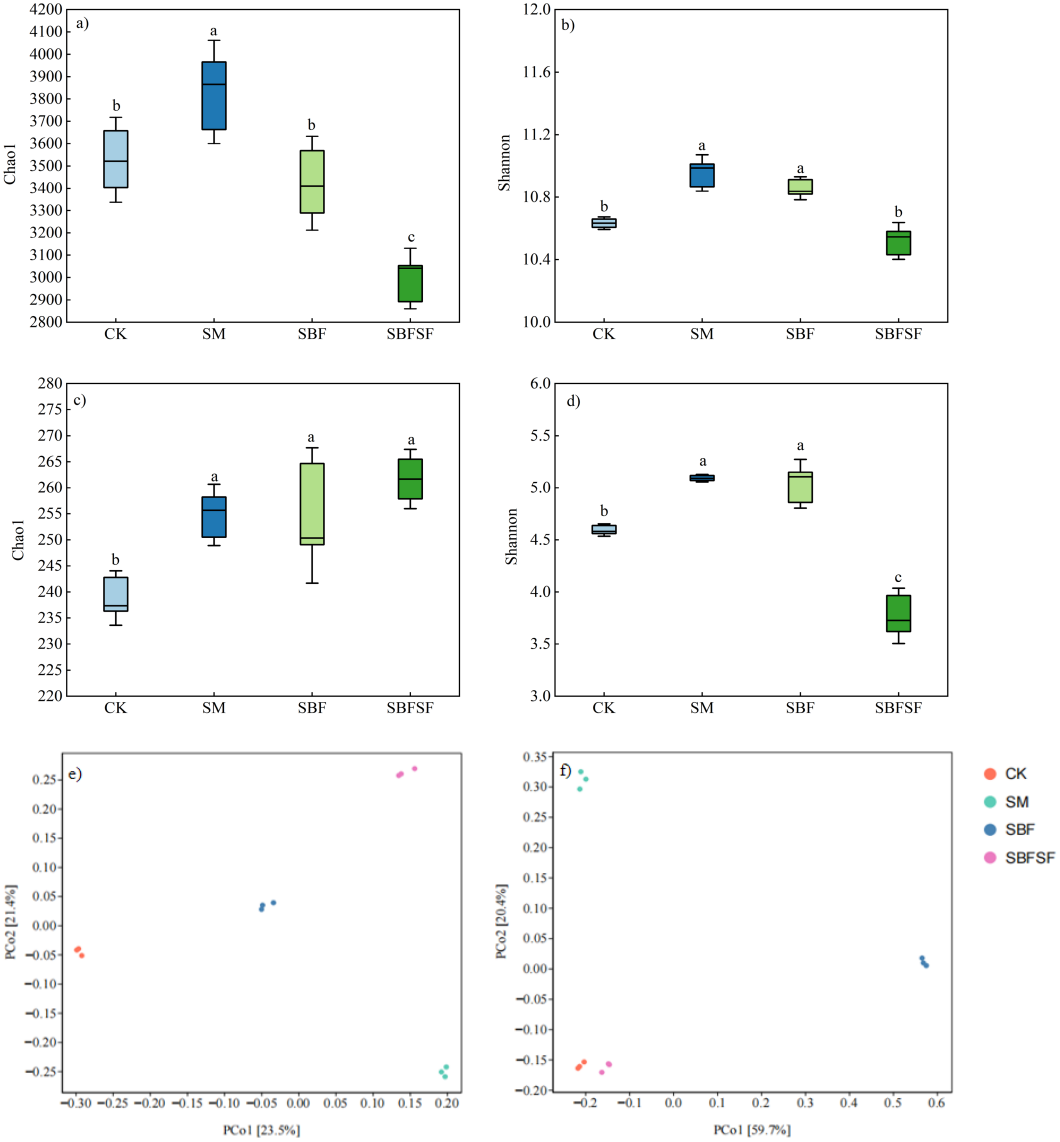
Soil bacterial and fungi diversity index (based on OTU level). **(a)** Chao 1 index of bacteria, **(b)** Shannon 1 index of bacteria, **(c)** Chao 1 index of fungi, **(d)** Shannon 1 index of fungi, **(e)** principal coordinate analysis (PCoA) of bacteria, **(f)** principal coordinate analysis (PCoA) of fungi.

SBFSF significantly increased only the fungal Chao1 index, while showing significant reductions in all other alpha-diversity indices for both bacterial and fungal communities. In contrast, SM exhibited significant increases across all measured alpha-diversity indices. Regarding SBF, no significant difference was observed in the bacterial Chao 1 index compared to CK, however, all remaining alpha-diversity indices showed statistically significant enhancement (Figures 5a–d).

The composition of bacterial and fungal communities in soil was different under various mulching treatments, as shown by the Principal Coordinates Analysis (PCoA). The first axis of variation for the bacterial community explained 23.5%, while the second axis explained 21.4% in community structure. The values for all of the mulching treatments had a clear separation from control (CK) (Figure 5e). In contrast, although the fungal communities under various mulching treatments did not overlap with CK in the ordination space, the SBFSF treatment was relatively closer to CK. The first and second axes explained 59.7 and 20.4% of the variance in fungal community composition, respectively (Figure 5f).

**Figure 6:**
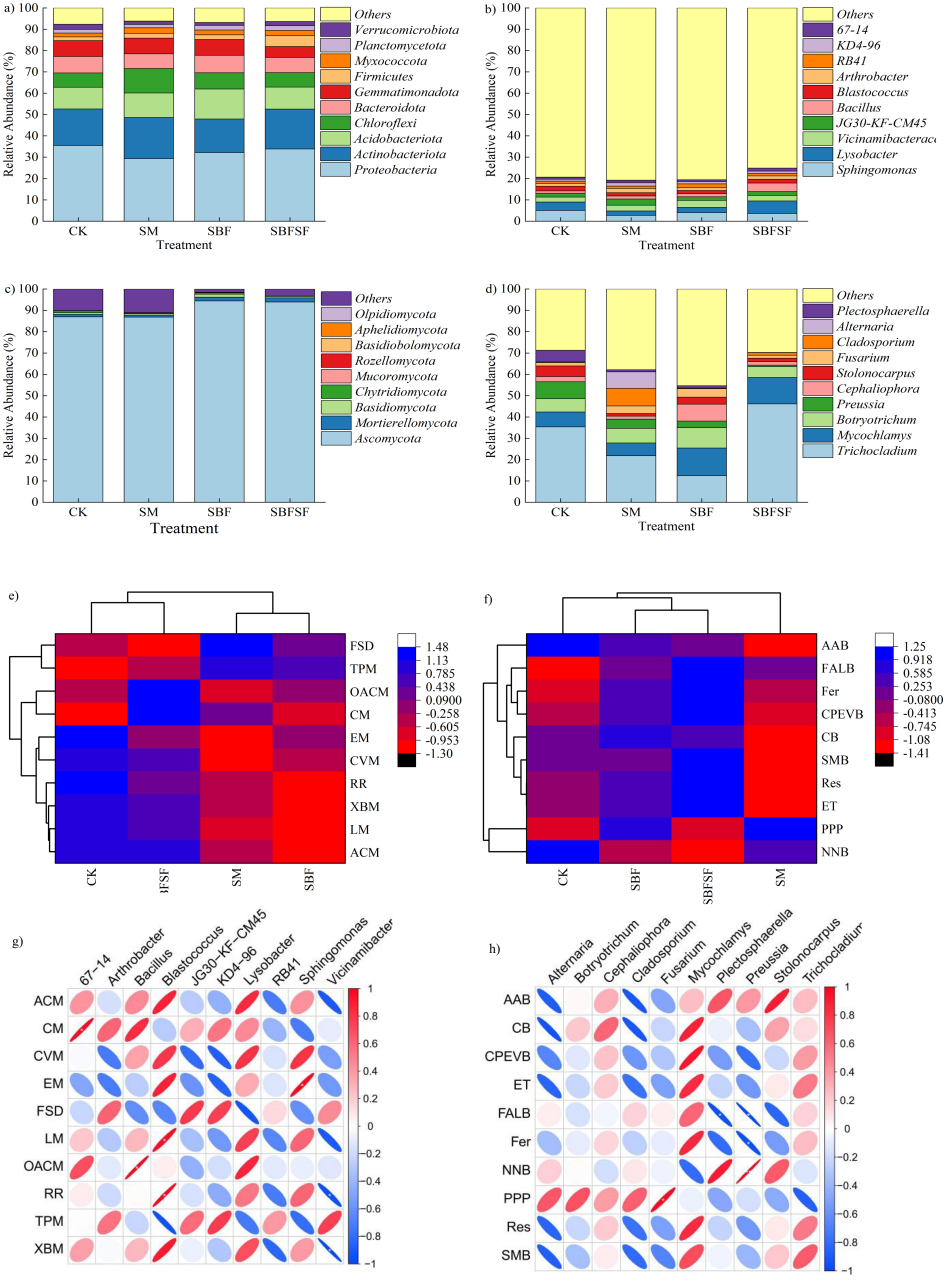
Relative abundance of bacterial communities at the phylum **(a)** and genus **(b)** levels, relative abundance of fungal communities at the phylum **(c)** and genus levels **(d)**, functional prediction and differences of bacterial **(e)** and fungal **(f)** communities under different mulching practices, correlation analyses between bacterial functions and genera **(g)**; correlation analyses between fungal functions and genera **(h)**. FSD, Folding, sorting and degradation; TPM, Metabolism of terpenoids and polyketides; OACM, Metabolism of other amino acids; CM, Carbohydrate metabolism; EM, Energy metabolism; CVM, Metabolism of cofactors and vitamins; RR, Replication and repair; XBM, Xenobiotics biodegradation and metabolism; LM, Xenobiotics biodegradation and metabolism; ACM, Amino acid metabolism; AAB, Amino acid biosynthesis; FALB, Fatty acid and lipid biosynthesis; Fer, Fermentation; CPEVB, Electron carrier, and vitamin biosynthesis; CB, Carbohydrate biosynthesis; SMB, Secondary metabolite biosynthesis; Res, Respiration; ET, Electron transfer; PPP, Pentose phosphate pathways; NNB, Nucleoside and nucleotide biosynthesis.

The composition and the relative abundance of soil bacteria were analyzed at both the phylum (top 10) and genus (top 10) levels. Compared to CK, SBFSF, SM and SBF significantly reduced the relative abundance of *Proteobacteria* by −17.20% to −4.60%. Compared to other treatments, SBFSF significantly increased the relative abundance of *Firmicutes* by +125.18 to 193.55%, while SM enhanced *Actinobacteria* (+2.63% to +22.04%) and *Chloroflexi* (+50.70–71.87%). SBF showed a significant increase in *Acidobacteria* (+21.79–37.12%) (Figure 6a). At the genus level, SBFSF enriched *Lysobacter* (+49.19% to +186.62%) and *Bacillus* (+168.56% to +273.00%), whereas reduced the relative abundance of *Sphingomonas* (−28.32% to −9.60%). SM increased *JG30-KF-CM45* (+59.61% to +73.40%) and *Arthrobacter* (+28.94% to +55.42%), whereas reduced the relative abundance of *Sphingomonas* (−46.51% to −25.37%). SBF led to higher relative abundance of *Vicinamibacteraceae* (+19.34% to +41.39%) (Figure 6b).

Fungal community analysis revealed that *Ascomycota* was the dominant phylum with relative abundances ranging from 86.76 to 94.38% (Figure 6c). Compared to other treatments, SBFSF significantly increased the relative abundances of *Ascomycota* (+7.99% to +8.19%) and *Mortierellomycota* (+11.05% to +98.71%). At the genus level, the relative abundance of *Trichocladium* in SBFSF was significantly higher than that in other treatments, showing an increase of 30.43 to 269.95%, while SM treatment notably enriched *Fusarium* (+120.39% to +149.16%), *Cladosporium* (587.22% to +4141.65%), and *Alternaria* (10953.15% to +32375.165). Moreover, SBF displayed significant increases in both *Cephaliophora* (+218.39% to +454.82%) and *Fusarium* (+12.48% to +180.25%) (Figure 6d).

Distinct soil microbial taxa enriched in various mulching treatments were identified through LEfSe analysis (Supplementary Figure S2). Overall, the numbers of indicator bacterial taxa associated decreased across all mulching types. Specifically, the SM and SBFSF management strategies promoted the enrichment of fungal taxa, while the SBF practice had a diminishing effect. Notably, the bacterial genus *Lysobacter* showed enrichment in response to SBFSF treatment, while *Chloroflexi* predominated under SM and *Acidobacteriota* under SBF. Among fungal taxa, *Trichocladium* was enriched in SBFSF, *Dothideomycetes* in SM, and *Microascales* in SBF.

Functional prediction of bacterial metabolism revealed distinct metabolic profiles in SBFSF compared to SM and SBF, with enhanced activities in amino acid metabolism, carbohydrate metabolism, metabolism of other amino acids, and lipid metabolism (Figure 6E). For fungal metabolic functions, SBFSF demonstrated increased capabilities in multiple pathways relative to SM and SBF, including electron transfer, respiration, secondary metabolite biosynthesis, cofactor and prosthetic group biosynthesis, electron carrier synthesis, vitamin biosynthesis, fermentation, as well as acid and lipid biosynthesis (Figure 6F).

At the bacterial genus level, *Bacillus* showed a significant positive correlation with metabolism of other amino acids, with a notably high correlation coefficient of 0.83 for carbohydrate metabolismz although not statistically significant. *Lysobacter* exhibited strong correlations with amino acid metabolism (0.85), lipid metabolism (0.76), and metabolism of other amino acids (0.86). Additionally, *67–14* showed a significant positive correlation with carbohydrate metabolism and a notably high correlation coefficient of 0.70 for other amino acid metabolism (Figure 6G). At the fungal genus level, *Mycochlamys* demonstrated substantial correlations with electron carrier and vitamin biosynthesis (0.92), electron transfer (0.84), respiration (0.84), and carbohydrate biosynthesis (0.88) (Figure 6H).

### 3.5 Relationship between microbial communities, soil properties, and tomato plant growth

**Figure 7:**
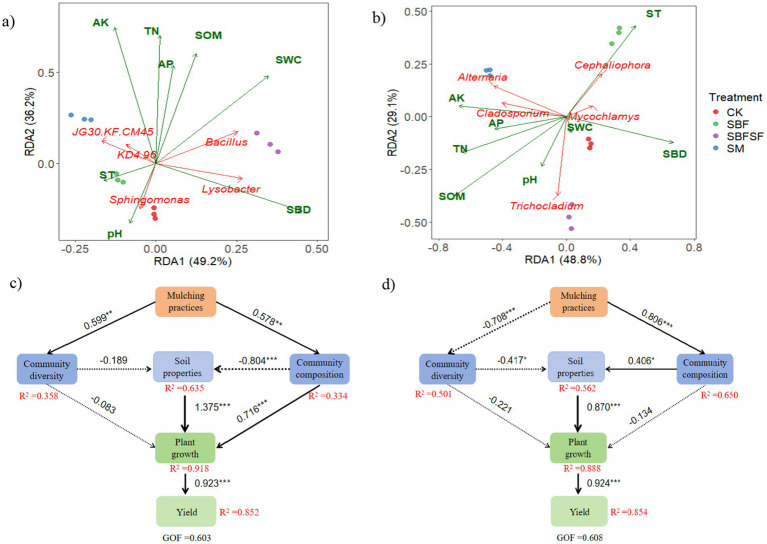
RDA of soil bacterial **(a)** and fungi **(b)** communities in relation to environmental factors, partial least squares path model (PLS-PM) direct and indirect effects of bacteria community **(c)**, fungal community **(d)** and mulching practices on tomato yield. Solid lines denote positive correlations and dashed lines denote negative correlations. Numbers above lines are the standardized path coefficients. GOF, goodness-of-fit. *, *p* < 0.050 level; **, *p* < 0.010 level; ***, *p* < 0.001 level. Bacterial community compositions suggest the *Proteobacteria* and *Actinobacteriota, Acidobacteriota, Chloroflexi, Bacteroidota*, respectively. Fungal community compositions suggest the *Ascomycota* and *Mortierellomycota*. Community diversity depict the Chao 1 and Shannon indices of the bacteria and fungi. Soil properties suggest the soil organic matter, total nitrigon, available potassium, available phosphorus, pH, soil bulk density, soil temperature and soil water content, respectively. Plant growth suggest the dry matter weight, root length, root surface area, respectively.

Redundancy analysis conducted at the bacterial genus level, focusing on the top five scoring bacteria, revealed that the two principal axes explained 49.2 and 36.2% of the total variance contribution, respectively (Figure 7a). Specifically, the application of SBFSF was found to enhance the proliferation of *Bacillus* and *Lysobacter*. *Bacillus* exhibited a significant positive correlation with soil water content (SWC) (*p* < 0.001) and a negative correlation with pH (*p* < 0.05). Moreover, the use of SM was observed to stimulate the growth of *JG30-KF-CM45*, which displayed a negative correlation with soil bulk density (SBD) (*p* < 0.05). Additionally, *Sphingomonas* showed negative correlations with available potassium (AK), available phosphorus (AP), total nitrogen (TN), and soil organic matter (SOM) (*p* < 0.01). Similarly, redundancy analysis conducted at the fungal genus level, focusing on the top five fungi, in relation to soil environmental factors, indicated that the two principal axes explained 48.8 and 29.1% of the total variance, respectively (Figure 7b). The application of SBFSF was found to promote the growth of *Trichocladium*, while SM significantly enhanced the growth of *Alternaria*. *Alternaria* exhibited a positive correlation with total nitrogen (TN) (*p* < 0.05) and a negative correlation with soil bulk density (SBD) (*p* < 0.01). Furthermore, the use of SBF was associated with increased growth of *Cephalophora*, which displayed negative correlations with soil organic matter (SOM), total nitrogen (TN), and available potassium (AK) (*p* < 0.05).

Further analysis using partial least squares path modeling (PLS-PM) revealed that a combination of soil bacterial community composition, diversity, soil properties, and plant growth accounted for 60.3% of the variability in tomato yield (Figure 7c). Similarly, soil fungal community composition, diversity, soil properties, and plant growth elucidated 60.8% of the variance in tomato yield (Figure 7d). Mulching practices exerted a direct and significant positive effect on soil bacterial community composition (path coefficient = 0.578, *p* < 0.010) and diversity (path coefficient = 0.599, *p* < 0.010), but only bacterial community composition had significant negative impacts on soil properties (path coefficient = −0.804, *p* < 0.001), which subsequently positively influenced tomato plant growth (path coefficient = 1.375, *p* < 0.001) and ultimately enhanced tomato yield (path coefficient = 0.923, *p* < 0.001). The mulching practices exerted a direct and positive effect on the composition of the soil fungal community (path coefficient = 0.806, *p* < 0.001), consequently positively influenced soil properties (path coefficient = 0.406, *p* < 0.050). Conversely, it negatively affected fungal diversity (path coefficient = −0.708, *p* < 0.001), which subsequently negatively affected soil properties (path coefficient = −0.417, *p* < 0.050). The improved soil properties then positively promoted tomato plant growth (path coefficient = 0.870, *p* < 0.001), ultimately benefiting tomato plant growth (path coefficient = 0.924, *p* < 0.001).

## 4 Discussion

### 4.1 Effect of different mulching practices on soil physicochemical properties

Previous studies has shown that both plastic film mulching and straw mulching are both able to prevent water movement and atmospheric gaseous exchange in soil (Chai et al., 2022; Zhang et al., 2021; Zribi et al., 2015), thus enhancing soil hydrothermal properties compared with bare plots. Our study found that all three mulching treatments enhanced soil water retention capacity, with SBFSF exhibiting the highest moisture retention due to the complementary functions of its components within a fully covered microenvironment. At 80 days after transplantation, no significant differences in soil water content were detected among the three mulching treatments. This result may be explained by the high transpiration rates of tomato plants under all mulching practices during this stage, leading to increased soil water depletion and consequently diminished variation in soil moisture levels.

Mulching practices enhanced soil temperature, with increases ranging between 0.01–2.21 °C at 10 cm depth and 0.02–2.05 °C at 20 cm depth, which is in agreement with former study results (Miao et al., 2025; Wang Y. et al., 2025; Qu and Feng, 2022). In this study, both SBFSF and SM demonstrated the cooling effect under high temperatures and warming effect under low temperatures, indicating that the soil temperature regulation effects of different mulching practices exhibited temporal variations. SBF exhibited the strongest warming effect in the early stage of tomato growth, but it could not regulate temperature during high-temperature period. In contrast, SBFSF maintained the most stable soil thermal conditions during the entire growth period, which implied combined effect of full soil coverage on decreasing temperature fluctuation. These results emphasized the superior performance of SBFSF in generating a balanced and flexible thermal environment and such characteristics might contribute to enhancing tomato growth and yield stability under dynamic climate.

Our study demonstrated that both SM and SBFSF enhanced rhizosphere soil fertility after 3 years of tomato cultivation by increasing the content of soil organic matter, total nitrogen, and available nutrients (Zhu et al., 2022). This improvement occurred because straw mulching on the soil surface releases nutrients through microbial decomposition (Liu et al., 2014), thereby enriching the topsoil nutrient pool—a finding consistent with the research of Biswal et al. (2022), Wang J. et al. (2021), and Chen et al. (2022). SM treatment reduced the relative abundance of *Sphingomonas*, and RDA analysis indicated that *Sphingomonas* was negatively correlated with soil available potassium, available phosphorus, and organic matter (*p* < 0.01). Therefore, it can be inferred that *Sphingomonas* played an important role in the soil fertility of SM treatment. The improvement in soil fertility under SBFSF was also associated with its regulation on soil temperature. Small fluctuation of soil temperature in return leads to the low mineralization rates of SOC and available nutrients (Zhou et al., 2012). We attribute the increased soil fertility under SBFSF to the combined regulatory effects of plastic film on ridges and straw in furrows. SBFSF likely produces a more favorable soil microenvironment which together support improved soil fertility. In addition, SM reduced soil bulk density which contributed to crop growth and roots residue input into soil for conversion into plant available nitrogen and phosphorous nutrient (Wang et al., 2024). Under SBF, initially higher soil temperatures enhance the process of organic matter decomposition and exhaustion of available nutrient resources at a faster rate, leading to lower rhizosphere fertility.

### 4.2 Effect of different mulching practices on soil microbial community diversity

Soil microorganisms are the most dynamic components of soil, with their community diversity and composition being influenced by mulching practices. In the present study, all mulching treatments significantly affected the richness and diversity of rhizosphere soil bacterial and fungal populations, and was due to the difference in soil temperature, moisture, and organic matter input by mulching practices (Luo et al., 2019; Ling et al., 2016; Chen et al., 2017). SBFSF significantly increased fungal Chao1 index and decreased all other alpha-diversity indices for both bacteria and fungi. This was an indication that although SBFSF could promote the richness of fungus, it might also act as a limited ecological niche and inhibit overall microbial diversity (Zhao et al., 2024). On the contrary, SM caused significant increases in all alpha-diversity measures which may indicate that a niche is less selective for richness and evenness. A majority of the previous reports suggested that straw mulching increased bacterial diversity (Zhang et al., 2024), while results on fungal communities have not been consistent (Fu et al., 2019; Liu et al., 2024) due to variation in source and time of straw application and soil type, climate condition. Soil bacteria and fungi diversity were significantly increased by SBF, partially in line with Dong et al. (2017). The increase in soil bacterial and fungal diversity under the SBF may be attributed to the enhanced heterogeneity of the soil micro-environment induced by plastic film mulching. Variations in temperature, moisture, and oxygen availability across different soil layers create distinct niches, which can support the proliferation of microorganisms occupying diverse ecological roles (Li et al., 2024).

In our study, SBFSF was effective in enhancing numbers of bacterial genera with biocontrol potential and nutrient cycling properties. The SBFSF enriched *Bacillus* and *Lysobacter*, which correlated with the soil carbon and nitrogen cycling. *Bacillus* are also well known and have been reported to possess a potential for antagonism against soil-borne pathogens, mediated by the production of antimicrobial compounds (Saxena et al., 2020), this in turn could enhance crop productivity and carbon sequestration (Wang et al., 2015). The predicted functional linkage to the metabolism of other amino acids strengthens the inference for their greater contribution to N cycling and the biosynthesis of bioactive compounds. Similarly, *Lysobacter* can also lyse fungi and are involved in the decomposition of resistant organic matter (Afoshin et al., 2020). Their predicted functional profiles, associated with amino acid metabolism, lipid metabolism, and metabolism of other amino acids, suggested their multifunctional potential in C and N turnover. These findings suggested that SBFSF build a disease-suppressive soil environment with continuous nutrient cycling. In contrast, SM significantly increased *Actinobacteriota* and *Chloroflexi* but also enriched potentially pathogenic fungi. It is well known that members of *Actinobacteriota* can degrade complex lignocellulose and produce antibiotic compounds, which suppress the growth and reproduction of the pathogenic microorganisms (Yang et al., 2024; Ma et al., 2018; Zheng et al., 2022), *Chloroflexi* involving organic substance decomposition, carbon cycling to increase soil conditions making it more suitable for plant growth (Hu et al., 2023). At the genus level, SM promoted the substantial growth of *JG30-KF-CM45*, suggesting that *JG30-KF-CM45* may play an important role in soil carbon and nitrogen cycling. However, the substantial increase in *Fusarium*, *Cladosporium*, and *Alternaria* - species all containing known pathogens causing various disorders of tomato like wilt, leaf spot and fruit rot (Zhao L. et al., 2023; Kong et al., 2023; Zhang et al., 2025)—were alarming with respect to the possible risk potential of diseases due to SM. Thus, while the effect of SM on beneficial decomposers such as *Actinobacteriota* and *Chloroflexi* are promoted, the increase in plant potential pathogens appears to counterbalance those beneficial ecological effects. SBF significantly enriched *Acidobacteria* and *Vicinamibacteraceae*, taxa commonly associated with acidic environments (Gonçalves et al., 2024), a finding consistent with the decreased soil pH observed under SBF in our study. Furthermore, SBF increased the relative abundance of *Fusarium* and *Cephaliophora*. Of particular note, a strain of *Cephaliophora* known to cause tomato leaf spot was first reported in the United States in 2023 (Luo et al., 2020), suggesting that long-term silver-black plastic film mulching may also promote the proliferation of potential pathogenic fungi. It is noteworthy that the identification of the aforementioned pathogens is based on an analysis of relevant literature, and their precise functions require further experimental validation.

Fungal community analysis revealed that SBFSF increased the abundance of *Ascomycota*, *Mortierellomycota* at the phylum level and *Trichocladium* at the genus level, which are involved in decomposition of cellulose and chitin (Li et al., 2020; Ozimek and Hanaka, 2021; Wu et al., 2022). More importantly, functional predictions indicated that SBFSF enhanced multiple metabolic pathways including electron transfer, respiration, vitamin biosynthesis, and secondary metabolite production. These improvements indicate a more metabolically active fungal community, which is capable to sustain soil ecosystem functioning.

The differing responses of microbial community to mulching practices indicates a balance between diversity and function maintenance. Even if SM and SBF promoted taxonomic diversity, SBFSF generated a functionally specialized community with greater metabolic potential and lower potential pathogen load. This is indicative that the functional attributes may be more important than overall diversity in supporting soil health and plant productivity.

### 4.3 Relationships between soil properties, microbial communities and tomato plant growth

Mulching is widely utilized in field crop and vegetable production and has been shown to promote crop growth (Sharma et al., 2023; Wang J. et al., 2025; Zhao Y. et al., 2023). Similarly, our study demonstrated that all mulching treatments significantly enhanced tomato yield compared to CK, with SBFSF exhibiting the most pronounced effect. These improvements can be attributed to the integrated modifications in plant growth and soil microenvironment induced by the different mulching practices.

The higher yield under SBFSF compared to SBF and SM was also consistent with enhanced dry matter accumulation and root growth. Increased root length and area under SBFSF probably contributed to enhanced nutrient and water uptake (Mooney et al., 2010; Guo et al., 2024) which resulted in higher biomass production and fruit yield. The early advantage in dry matter under SBF at 20 days may be attributed to its more effective soil warming during initial growth. However, SBFSF surpassed all other treatments by 40 days, indicating that its combined regulation of temperature and moisture better sustained plant development. Furthermore, SBFSF promoted the proliferation of beneficial soil microorganisms such as *Firmicutes*, *Bacillus*, *Lysobacte*, *Ascomycota*, *Mortierellomycota* and *Trichocladium*, consequently, the beneficial microorganisms improved key soil properties, including soil organic matter content, total nitrogen, and available nutrients, ultimately leading to an increasing in tomato production (Tao et al., 2024). These findings align with previous reports demonstrating that microbial community modulation can enhance crop productivity (Wang et al., 2024; Li et al., 2020). Conversely, the stimulation of potential pathogenic species (*Fusarium*, *Alternaria*, *Cladosporium*) under SM may have partially counteracted the benefits of nutrient availability, potentially explaining why tomato production under SM underperformed compared to SBFSF although they had improved some soil properties. Similarly, the SBF treatment also elevated the abundance of potentially pathogenic fungi, including *Cephaliophora* and *Fusarium*. Given its suboptimal soil physicochemical performance, this likely contributed to the reduced yield of SBF compared to SBFSF. Based on partial least squares path modeling (PLS-PM), it suggested that soil properties were strongly and directly associated with tomato growth, whereas mulching practices were linked to enhanced tomato growth primarily through their association with changes in bacterial and fungal diversity and community structure, which in turn were related to soil properties.

Our study revealed an increase in the population of soil potential pathogenic fungi following 3 years of plastic film mulching alone and straw mulching alone. Despite this increase, tomato yields remained higher compared to the control. Thus, the potential continuous decrease in tomato yield over successive planting years warrants further observation over an extended period. Our study spanned a limited duration of 3 years; moving forward, our research will persist in examining both tomato soil quality and yield. Additionally, we examined soil nutrient and microbial conditions only before tomato vine removal and did not track their dynamics across multiple time points. Investigating those temporal changes is a direction for our future research.

## 5 Conclusion

This three-year study demonstrates that film-straw dual mulching (SBFSF) is the most effective practice among the treatments tested, enhancing tomato yield by optimizing soil moisture, temperature, and nutrient availability. Its superior performance is primarily attributed to the selective enrichment of beneficial functional microorganisms, such as plant growth-promoting rhizobacteria and key taxa involved in carbon-nitrogen cycling, despite reducing overall microbial diversity. In contrast, single mulching practices promoted certain potential pathogenic fungi. The PLS-PM model indicated that mulching directly shape the microbial community, which promotes tomato growth by indirectly altering soil properties. Although the specific contributions of film versus straw warrant further investigation (e.g., using fully covered single-material controls), the SBFSF system as a whole is advocated as a sustainable agricultural strategy for enhancing tomato productivity via the promotion of a beneficial soil micro-ecosystem.

## Data Availability

The datasets presented in this study can be found in online repositories. The names of the repository/repositories and accession number(s) can be found here: https://ngdc.cncb.ac.cn/gsa/browse/CRA037497.
