# Supplementary material for: Effects of different mulching practices on soil properties and soil microbial communities in tomato production
Source: Front Microbiol. 2026 Feb 3;16:1734062. doi: 10.3389/fmicb.2025.1734062 (PMC12912733; doi:10.3389/fmicb.2025.1734062)
Supplement: Supplementary file 1 [file Table_1.docx]

**Table S1** Field application of pesticides for pest and disease control

| Disease/Pest | Pesticide Name | Application Method | Frequency of use |
| --- | --- | --- | --- |
| Early blight | 10% Difenoconazole water-dispersible granules | 1500× dilution, foliar spray | ≥7 d |
| Late blight | 250 g/L Pyraclostrobin emulsifiable concentrate | 1000-1500× dilution, foliar spray | 7-10d |
| Aphids and whiteflies | 70% Imidacloprid water-dispersible granules | 5000× dilution, foliar spray | ≥3d |

**Table S2** Correlation Analysis Between Soil Moisture and Temperature (Pearson analysis)

| Days After Tomato Transplanting | 20D | 40D | 60D | 80D | 110D |
| --- | --- | --- | --- | --- | --- |
| Soil moisture and temperature correlation Coefficient（r） | 0.304 | 0.294 | 0.575 | 0.544 | 0.966 |
| Significance (*p*-value) | >0.01 | >0.02 | >0.03 | >0.04 | <0.001 |
