# Supplementary material for: Effects of different mulching practices on soil properties and soil microbial communities in tomato production
Source: Front Microbiol. 2026 Feb 3;16:1734062. doi: 10.3389/fmicb.2025.1734062 (PMC12912733; doi:10.3389/fmicb.2025.1734062)
Supplement: Supplementary file 2 [file Data_Sheet_1.docx]

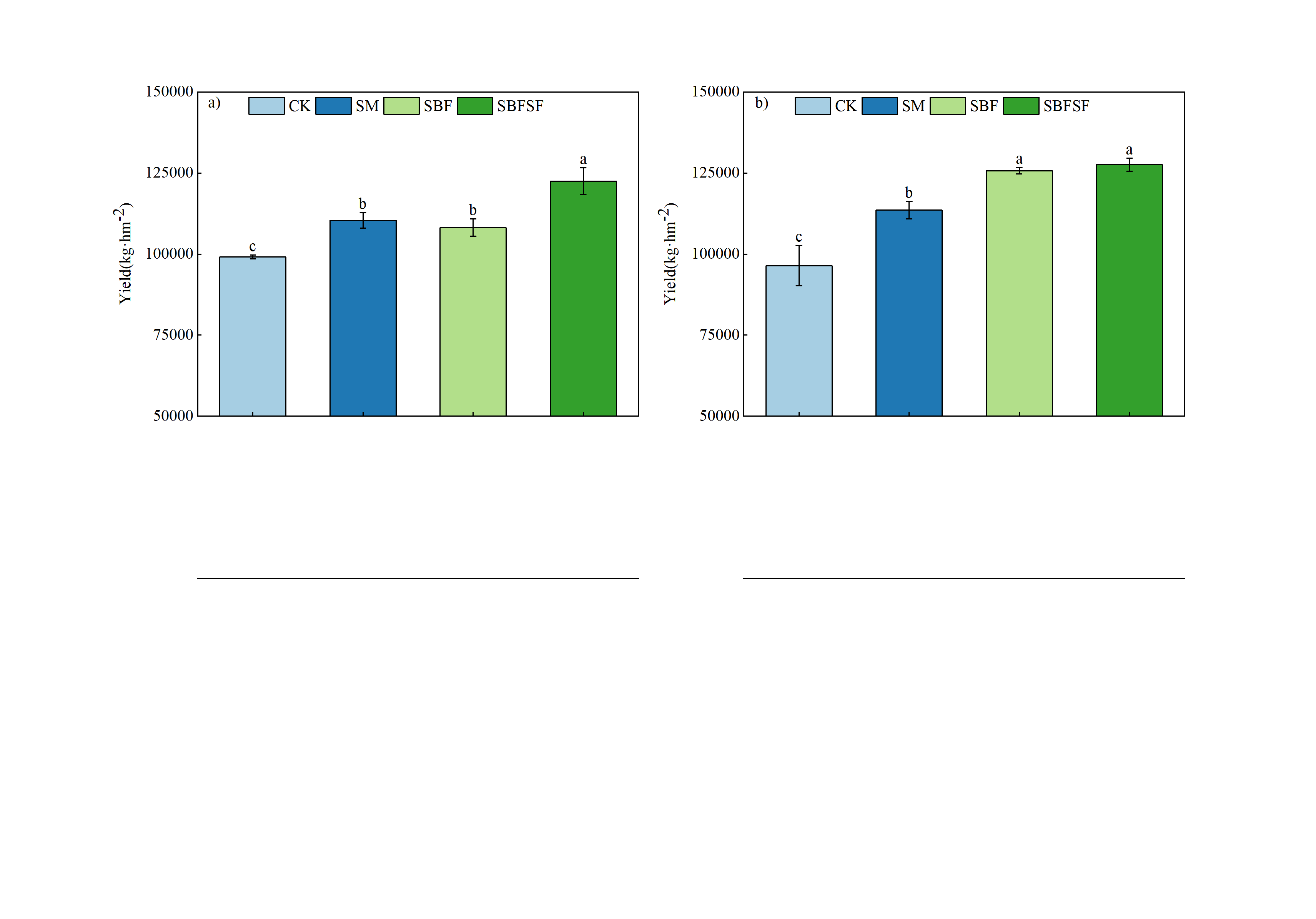


**Figure. S1** Tomato yield in 2022 (a) and 2023 (b).

Data are presented as the mean ± standard deviation (SD) (n = 3). Different lowercase letters above the bars indicate significant differences among treatments according to Duncan’s test (p < 0.05). CK, no mulch; SM, straw mulching; SBF, plastic film mulching; SBFSF, combined film and straw mulching.

**Figure. S2**  Abundance and composition of biomarker bacteria(a) and fungi (b) communities in different mulching practices.


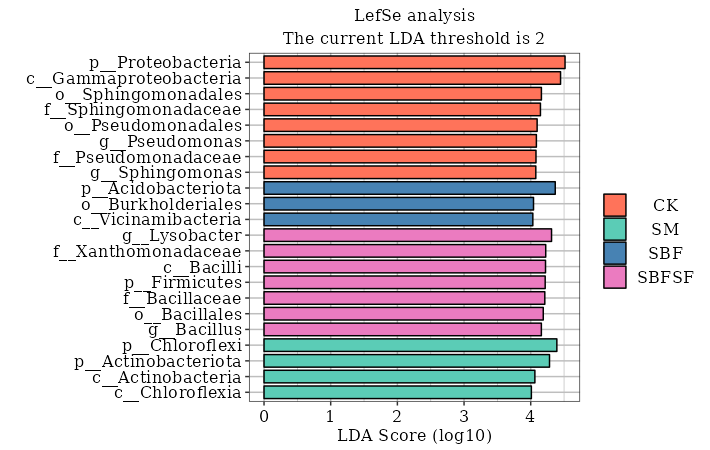

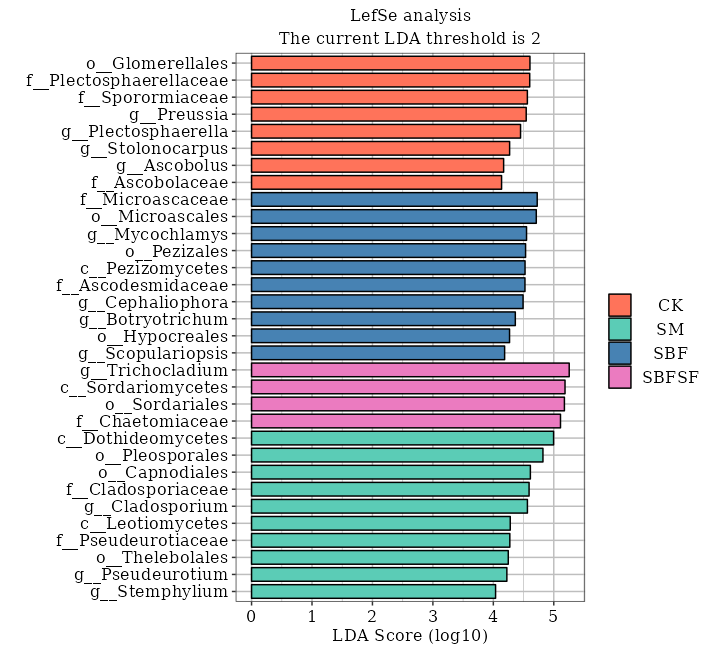


a)

b)
